# Expression of maternally derived *KHDC3, NLRP5, OOEP* and *TLE6* is associated with oocyte developmental competence in the ovine species

**DOI:** 10.1186/s12861-014-0040-y

**Published:** 2014-11-25

**Authors:** Daniela Bebbere, Federica Ariu, Luisa Bogliolo, Laura Masala, Ombretta Murrone, Mauro Fattorini, Laura Falchi, Sergio Ledda

**Affiliations:** Department of Veterinary Medicine, University of Sassari, via Vienna 2, 07100 Sassari, Italy

**Keywords:** Ovine embryo, Oocyte developmental competence, Subcortical Maternal Complex

## Abstract

**Background:**

The sub-cortical maternal complex (SCMC), located in the subcortex of mouse oocytes and preimplantation embryos, is composed of at least four proteins encoded by maternal effect genes: OOEP, NLRP5/MATER, TLE6 and KHDC3/FILIA. The SCMC assembles during oocyte growth and was seen to be essential for murine zygote progression beyond the first embryonic cell divisions; although roles in chromatin reprogramming and embryonic genome activation were hypothesized, the full range of functions of the complex in preimplantation development remains largely unknown.

**Results:**

Here we report the expression of the SCMC genes in ovine oocytes and pre-implantation embryos, describing for the first time its expression in a large mammalian species.

We report sheep-specific patterns of expression and a relationship with the oocyte developmental potential in terms of delayed degradation of maternal SCMC transcripts in pre-implantation embryos derived from developmentally incompetent oocytes.

In addition, by determining *OOEP* full length cDNA by Rapid Amplification of cDNA Ends (RACE) we identified two different transcript variants (*OOEP1* and *OOEP2*), both expressed in oocytes and early embryos, but with different somatic tissue distributions.

*In silico* translation showed that 140 aminoacid peptide OOEP1 shares an identity with orthologous proteins ranging from 95% with the bovine to 45% with mouse. Conversely, *OOEP2* contains a premature termination codon, thus representing an alternative noncoding transcript and supporting the existence of aberrant splicing during ovine oogenesis.

**Conclusions:**

These findings confirm the existence of the SCMC in sheep and its key role for the oocyte developmental potential, deepening our understanding on the molecular differences underlying cytoplasmic vs nuclear maturation of the oocytes.

Describing differences and overlaps in transcriptome composition between model organisms advance our comprehension of the diversity/uniformity between mammalian species during early embryonic development and provide information on genes that play important regulatory roles in fertility in nonmurine models, including the human.

**Electronic supplementary material:**

The online version of this article (doi:10.1186/s12861-014-0040-y) contains supplementary material, which is available to authorized users.

## Background

The subcortical maternal complex (SCMC) is a multi-protein complex located in the sub-cortex of mouse oocytes and pre-implantation embryos [1] composed by at least four proteins: Oocyte Expressed Protein [(OOEP) also known as Factor Located in Oocytes Permitting Embryonic Development (FLOPED)], NLR family, pyrin domain containing 5 [(NLRP5) also known as Maternal Antigen That Embryo Requires (MATER)], Transducin-Like Enhancer of Split 6 (TLE6) and KH domain-containing protein 3 [(KHDC3), also known as FILIA)]. The SCMC was seen to be essential for murine zygote progression beyond the first embryonic cell divisions [1], however little is known about the complex in species other than mouse.

The proteins that contribute to the SCMC are encoded by maternal effect genes (MEGs; [1-4]). MEGs code for a special class of maternal transcripts necessarily required for the early cleavage events post fertilization [5,6]. They are expressed exclusively in oocytes and early embryos and are usually degraded by the time of embryonic genome activation (EGA), without compensation by embryonic transcription. Functional studies in mice have indeed demonstrated that the knockout of MEGs results in the inability of the embryo to develop beyond the first cleavage stage [2,7]. This was observed also in *Nlrp5*, *Ooep* and *Khdc3* case: the lack of either gene in mouse oocytes does not affect folliculogenesis, ovulation, or fertilization, but leads to the failure of early embryos to complete cleavage stage development, resulting in a striking female sterile phenotype in these mutant mice [1,2,4,8]. MEGs are involved in folliculogenesis, fertilization and pre-implantation embryo development [5], however their specific functions are often unclear, as in SCMC case.

The molecular mechanisms contributing to oogenesis and to early embryo development are highly conserved. In mammals, maternally-deposited transcripts were seen to be generally more conserved than the newly synthesized by the nascent embryo [9]; comparative genomics studies between mammals and phylogenetically distant chordates such as *X. laevis* and sea squirt *Ciona intestinalis* highlighted the conservation of the majority of the genes expressed in the oocytes [10-12].

Nevertheless, inherent species-specific differences, such as the ovulation quota and number of embryonic cell cycles required for EGA [13], exist between the traditional animal model (polyovulatory mouse) versus monoovulatory species, such as large domestic animals and primates, including humans. Numerous examples suggest that oocyte specific genes may not have identical functions in different species [14-19]. This is possibly related to the fact that several genes specifically expressed by the oocyte are organized in clusters and subject to rapid molecular diversification, often via gene duplication mechanisms [10,20-22]; this was seen to happen more often for genes that play a role in reproduction processes [23].

The picture of the oocyte transcriptome reveals a delicate balance between novelty and conservation. Comparative studies in nontraditional model systems are valuable to address dissimilarities and overlaps in transcriptome composition between model organisms, and provide information on genes that may play important regulatory roles in fertility in nonmurine models, including the human.

The SCMC appears to be an interesting combination between conservation and novelty: while *NLRP5/MATER* is conserved in several mammalian species, with a moderate grade of sequence homology [2,24-26], *OOEP* e *KHDC3* belong to an eutherian oocyte and embryo-expressed gene family subject to rapid molecular diversifications [27], whose expression analysis in oocytes and embryos of species other than mouse is still lacking.

In light of the above, the goal of the present study was to analyse the expression of the MEGs encoding the SCMC in the ovine species. We have previously studied the expression of *NLRP5* in sheep oocytes and embryos [24]. In the present work, *OOEP*, *KHDCL*3 and *TLE6* existence was assessed, and the expression pattern during *in vitro* oocyte maturation and embryo development was analysed, together with the potential expression in somatic tissues. A possible association between the expression of the SCMC four components and the oocyte developmental ability was assessed in a model of differential competence consisting of oocytes derived from adult (Ad) or prepubertal (Pr) donors.

Finally, the full length cDNA of *OOEP* was determined by Rapid Amplification of cDNA Ends (RACE).

## Methods

All chemicals in this study were purchased from Sigma Chemical CO. (St. Louis, MO, USA) unless otherwise stated.

All experiments involving animals were performed in accordance with the relevant guidelines for the care and use of animals. No animals were specifically generated or sacrificed for this study. Samples were obtained from animals regularly slaughtered in local abattoirs for food production purposes. All procedures were approved by the ethics committee of the University of Sassari, Italy.

### Oocyte recovery and in vitro maturation

Oocytes were recovered from the ovaries of prepubertal (30–40 days of age, body weight 6–10 kg) and adult (4–5 years of age, body weight 35–40 kg) Sarda sheep collected at a local slaughterhouse and transported to the laboratory within 1 hr in Dulbecco’s phosphate buffered saline (PBS) with antibiotics. After washing in fresh medium, ovaries were sliced using a micro-blade and the follicle content was released in TC-199 medium (with Earle’s salts and bicarbonate) supplemented with 25 mmol HEPES, 0.1 g/L penicillin, 0.1 g/L streptomycin, and 0.1% (w/v) polyvinyl alcohol.

The cumulus oocyte complexes (COCs) showing several intact cumulus cell layers and a compact cytoplasm were selected and matured in vitro in TCM 199 supplemented with 10% heat treated oestrus sheep serum (OSS), 0.1 IU/ml FSH and 0.1 IU/ml LH (Pergonal, Serono Italy) and 100 μM cysteamine. Groups of 30–35 COCs were cultured for 24 h in a humidified atmosphere of 5% CO_2_ in air at 38.5°C in four-well Petri dishes (Nunclon; Nalge Nunc, Roskilde, Denmark) with 600 μL maturation medium, layered with 300 μL mineral oil.

### In vitro fertilization and embryo development

Frozen–thawed spermatozoa of one single ram of proven fertility (Sarda breed, 4 years old, body weight 55–60 kg), selected by the swim-up technique, were used for all in vitro embryo production experiments. Two straws were thawed per IVF. The semen was washed twice with SOF medium with 2% (v/v) OSS for 20 minutes. Finally, sperm pellet (1 × 10^6^ spermatozoa mL^−1^) and IVM oocytes were incubated in fertilization medium [Synthetic Oviductal Fluid (SOF) medium with 2% OSS, 1 μg/mL heparin and 1 μg/mL hypotaurine] for 22 h at 38.5°C in an atmosphere of 5% CO_2_ and 5% O_2_ in N_2_ in four-well Petri dishes (Nunclon) [28].

Thereafter, presumptive zygotes were transferred and cultured for 8 days in four-well Petri dishes containing SOF + essential and non-essential amino acids at oviductal concentrations [29] +0.4% bovine serum albumin (BSA) under mineral oil, in a maximum humidified atmosphere with 5% CO_2_, 5% O_2_ and 90% N_2_ at 38.5°C [30]. The first cleavage was recorded between 24 and 26 h after fertilization.

### Gene expression analysis

#### Oocyte and embryo collection for gene expression analysis

The RNA samples were isolated from pools of denuded germinal vesicles (GV) and IVM metaphase II (MII) oocytes, and from pools of in vitro matured, fertilized and cultured (IVMFC) embryos at the two- (2C), four- (4C), eight- (8C), and 16-cell (16C) stage. Oocytes and embryos were obtained during several IVM, IVF and IVC sessions.

For a proper collection of oocytes, the assessment of nuclear maturation (GV or MII) was performed by visualization with a stereomicroscope (Olympus ZX40) at 40X magnification. Although the assessment of the meiotic stage cannot be performed with an absolute confidence in absence of staining, according to our experience, ovine oocytes soon after follicular collection are prevalently at the GV stage (98%), while they reach the MII stage and show the presence of the polar body after 24 h of in vitro culture. Oocytes at the GV and MII stages were denuded with a fine narrow bore pipette and defined as good quality when showing uniform cytoplasm.

Samples were analysed in pools, where every single pool contained 10 oocytes or 10 embryos, in order to obtain enough RNA for the analysis of the 7 transcripts. Five pools were analysed for GV (n = 5 pools) and MII (n = 5 pools) oocytes, while three pools were analysed for embryos at all developmental stages. Each pool was added to 2 μl RNase free water, snap frozen in liquid nitrogen, and stored at – 80°C until RNA isolation.

The same sampling was performed for oocytes and embryos derived from adult (Ad) and from prepubertal donors (Pr).

As a control for IVF procedure, a pool of embryos in each IVF run was cultured until the blastocyst stage to assess the developmental competence of the fertilized eggs. Only embryos collected from controlled experiments with rates of development to blastocyst stage of >25% (day 7) were used in the analysis.

Ten single blastocysts derived from Ad (n = 5) and Pr (n = 5) oocytes were analysed in order to assess the presence of the transcripts after the activation of the embryo genome.

### RNA Isolation and reverse transcription

Total RNA was isolated from the groups of oocytes or embryos with the RNeasy Micro Kit (Qiagen, Hilden, Germany) following manufacturer’s instructions. Five pg of luciferase mRNA (Promega) were added to each group prior to RNA extraction to account for RNA loss during the isolation process.

During the procedure, RNA was treated with DNase I to exclude any potential genomic DNA contamination. Isolated RNA was eluted in 15 μL RNase-free water and 13.5 μL immediately used for reverse transcription–polymerase chain reaction (RT-PCR).

One point five μL RNA was analysed with Agilent 2100 Bioanalyzer (Agilent Technologies) with an RNA 6000 Pico kit (Agilent Technologies), specifically designed for analysis of low concentrated RNA samples (analysis range of total RNA: 50–5000 pg/μl).

Reverse-transcription was performed in a final volume of 20 μL, consisting of 50 mM Tris–HCl (pH 8.3), 75 mM KCl, 3 mM MgCl_2_, 5 mM DTT, 1 mM dNTPs, 2.5 μM random hexamer primers, 0.05 μg oligo (dT)_18_ primers, 20 U RNase OUT and 100 U SuperScript III RT (all purchased at Invitrogen Corporation, Carlsbad, CA). The reaction tubes were incubated at 25°C for 10 min, then at 42°C for 1 h and finally at 70°C for 15 min to inactivate the reaction. One tube without RNA and one with RNA, but without reverse transcriptase, were analysed as negative controls. To quantify the mRNA recovery rate, 5 pg of luciferase mRNA (not subjected to RNA isolation) were subjected to cDNA synthesis as well.

### Real time-polymerase chain reaction

Primers for all genes studied are listed in Table 1. Relative quantification of transcripts was performed by real-time polymerase chain reaction (RT-PCR) in a 7900HT Fast Real-Time PCR System (Applied Biosystems). The PCR was performed in a 15 μL reaction volume containing 7.5 μL 2× SYBR Green PCR Master Mix (Applied Biosystems, Foster City, CA), 200 nM of each primer and cDNA equivalent to 0.25 oocytes or embryos.

**Table 1:** **Primers used for real-time PCR experiments**

**Gene**	**GenBank accession n.**	**Primers**	**Annealing temperature**	**Size (bps)**
***KHDC3***	XM_004011872	F: 5′ CAGACCCTGCTTCACGTTCA 3′	60°C	150
		R: 5′ CTTCTCAGAGCTTCGCGCC 3′		
***NLRP5***	HM037368.1	F: 5′ CAGCCTCCAGGAGTTCTTTG 3′	59°C	212
		R: 5′ GACAGCCTAGGAGGGTTTCC 3′		
***OOEP 1***	KF218578	F: 5′ ATCCGCTGGTGTTCTTCCTG 3′	60°C	149
		R: 5′ GAACACGGTGACTTCGACCA 3′		
***OOEP 2***	KF741040	F: 5′ TCCCCAAACTCCTTGCAGTG 3′	60°C	114
		R: 5′ CGGCAGGTAGGTGTCTGAAT 3′		
***TLE6***	XM_004009373	F: 5′ TACCTGCGCACCTGCCTGCT 3′	58°C	195
		R: 5′ ATTGGTGAAGCCAGCAAAAG 3′		
***ACTB***	NM_001009784	F: 5′ TTCCTGGGTATGGATCCTG 3′	60°C	162
		R: 5′ GGTGATCTCCTTCTGCATCC 3′		
***luciferase***	AF093685	F: 5′ GCTGGGCGTTAATCAGAGAG 3′	58°C	151
		R: 5′ GTGTTCGTCTTCGTCCCAGT 3′		

The PCR protocol consisted in two incubation steps (50°C for 5 min and 95°C for 2 min), followed by 40 cycles of amplification program [95°C for 15 s, gene-specific annealing temperature (see Table 1) for 30 s and 72°C for 30 s], a melting curve program (65–95°C, starting fluorescence acquisition at 65°C and taking measurements at 10-s intervals until the temperature reached 95°C) and finally a cooling step to 4°C. Fluorescence data were acquired during the 72°C extension steps.

To minimise handling variation, all samples to be compared were run on the same plate using a PCR master mix containing all reaction components apart from the sample.

The PCR products were analysed by generating a melting curve to check the specificity and identity of the amplification product. For each primer pair, the efficiency of the PCR reaction was determined by building a standard curve with serial dilutions of a known amount of template, covering at least 3 orders of magnitude, so that the calibration curve’s linear interval included the interval above and below the abundance of the targets. Only primers achieving an efficiency of reaction between 90 and 110% (3.6 > slope >3.1) and a coefficient of determination r^2^ > 0.99 were used for the analysis.

The sizes of the RT-PCR products were further confirmed by gel electrophoresis on a 2% agarose gel stained with Sybr Safe (Invitrogen) and visualised by exposure to blue light. The PCR products were sequenced (Model 3130 xl Genetic Analyzer; Applied Biosystems, Foster City, CA, USA) after purification with MinElute PCR purification kit (Qiagen) and sequence identities were confirmed with BLAST (http://www.ncbi.nlm.nih.gov/BLAST/). The relative quantification of the SCMC transcripts was performed after normalization to the number of oocytes and embryos [31-33]. Real-time RT-PCR data are presented as ΔCq, mean ± SEM. The relative amount of the target mRNA of each specific sample was obtained after subtraction of the calibrator expression level (ΔCT sample = CT sample - CT calibrator), where the calibrator was the sample showing the highest abundance (in most cases a pool of oocytes at the GV stage).

### Tissue collection, RNA isolation and reverse transcription

Ovine tissue samples including adult lung, muscle, kidney, cerebellum, testis, ovary, spleen, liver and heart were collected at a local slaughterhouse. All samples were immediately plunged into RNALater (Qiagen, Hilden, Germany) and stored at −80°C until RNA isolation. Total RNA was isolated using TRIzol reagent (Invitrogen, Carlsbad, CA) and treated with DNase I (Invitrogen, Carlsbad, CA) according to manufacturer’s protocols. Resulting RNA quantity and purity was spectroscopically checked with NanoDropLite (Fisher Scientific S.A.S., France).

Five hundred ng total RNA from each different tissue were reverse transcribed in a 20 μL reaction with 50 mM Tris HCl (pH 8.3), 75 mM KCl, 3 mM MgCl_2_, 5 mM DTT, 1 mM dNTPs, 2.5 μM Random Hexamer primers, 0.05 μg oligo (dT)_18_ primers, 20 U of RNase OUT™ and 100 U of SuperScript™ III RT (all provided by Invitrogen Corporation, Carlsbad, CA). Negative control reactions (without the enzyme) were carried out to confirm the absence of genomic DNA contamination. The reaction tubes were incubated at 25°C for 10 min, at 42°C for 1 h and finally at 70°C for 15 min to inactivate the reaction.

### Semi-quantitative PCR to analyse the transcript tissue distribution

Semi-quantitative PCR experiments were carried out to assess the expression of the four genes in the different somatic tissues. First-strand cDNA (~50 ng RNA) was used as a template for PCR amplification using gene-specific primers [see Table 1 – primer sequence, annealing temperature (Ta) and length of amplification products]. The PCR consisted in 5 min at 95°C, followed by 35 cycles of 94°C for 30 sec, Ta for 45 sec and 72°C for 30 sec, and a final extension at 72°C for 10 min. Ovine actin B (*ACTB*) gene was used as a positive control*.* The PCR products were evaluated by gel electrophoresis on a 2% agarose gel stained with Sybr Safe (Invitrogen) and visualised by exposure to blue light. The approximate size of the amplicons was estimated by comparison with the marker TrackIt φX174RF DNA/HaeIII.

### Cloning of ovine *OOEP* cDNA by PCR and RACE

Rapid Amplification of cDNA Ends (RACE) was performed to isolate the *OOEP* full length transcript, using the primer SMART (CACACACAATTAACCCTCACTAAAGG) and an oligo dT primer modified for retro transcription (CCTCTCTATGGGCAGTCGGTGATCCTCAGC(T)_21_. The product of the reverse-transcription was used as template for the following amplifications: to isolate the 5′ region, the primers Fsmart (CACACACAATTAACCCTCACTAAAGG) and Rgsp3OOEP (GAGAAGCAAATATCCTTAAATCTCTGCCT) were used, while the primers OOEPforB (CTGTTGCATGAATGTTGTCG) and RoligoCODA (CTCTATGGGCAGTCGGTGA) were employed to isolate the 3′ region.

Initially, the primers were designed based on conserved regions of bovine (NM_001077869), human (NM_001080507) and mouse (NM_026480) sequences, since the predicted ovine sequence (XM_004011571,) currently found in NCBI website, was not available when the analysis started.

TA Cloning kit (Invitrogen Corporation) was used to clone the fragments into the vector pCR2.1, using the chemocompetent cells TOP10. Several clones containing the fragments were sequenced.

The RACE was performed on total RNA isolated from prepubertal ovaries by the MICROCRIBI service at the Università degli Studi di Padova, Italy.

### Statistical analysis

Data were analysed with MINITAB Release 12.1 software package (Minitab Inc., State College, PA, USA). After testing for normality and equal variance using the Kolmogorov–Smirnov and Levene tests, respectively, transcript data were analysed with analysis of variance (ANOVA). Student t-test was conducted to evaluate differences between the expression levels of adult (Ad) vs prepubertal (Pr) oocytes and embryos (within each stage separately). ANOVA was conducted to evaluate differences between different stages of development in each class of embryos (adult and prepubertal separately).

Differences were considered significant when P <0.05.

## Results

### RNA in oocytes and pre-implantation embryos

RNA samples isolated from pools of 10 oocytes/embryos were analysed with Agilent 2100 Bioanalyzer (Agilent Technologies) with an RNA 6000 Pico kit (Agilent Technologies), specifically designed for analysis of low concentrated RNA samples (analysis range of total RNA: 50–5000 pg/μl). Results are described in Table 2. RNA samples isolated from 4-, 8- and 16- cell stage embryos are not included because below the lower limit of analysis (<50 pg/μl).

**Table 2:** **Total RNA content of ovine Ad and Pr oocytes and embryos**

**Stage**	**Mean concentration (pg/μl)**	**Mean SE (pg/μl)**	**Mean RIN value**	Isolated RNA (pg) per oocyte or embryo*	Estimated RNA (pg) per oocyte or embryo**
**GV Ad**	314	41.6	8.3	471	942
**GV Pr**	170	61	5.9	253	538
**MII Ad**	95	53	6.7	142	285
**MII Pr**	80	22	5	119	238
**2 cell Ad**	58	6	3.5	86	185
**2 cell Pr**	80	14	4.4	120	240

RNA samples isolated from pools of 10 oocytes/embryos were analysed with Agilent 2100 Bioanalyzer (Agilent Technologies) with an RNA 6000 Pico kit (Agilent Technologies), specifically designed for analysis of low concentrated RNA samples (analysis range of total RNA: 50–5000 pg/μl). RNA samples isolated from 4- 8- and 16- cell stage embryos are not included because below the lower limit of analysis (<50 pg/μl).

*Mean quantity of total RNA isolated per oocyte or embryo.

**Estimated RNA per oocyte or embryo, taking into account the RNA loss during RNA isolation, as resulted from analysis of the exogenous luciferase mRNA.

The quantification of the exogenous luciferase mRNA evidenced an RNA recovery rate of 52 ± 5% (Mean ± SD).

### cDNA cloning of ovine *OOEP*

**Figure 1:**
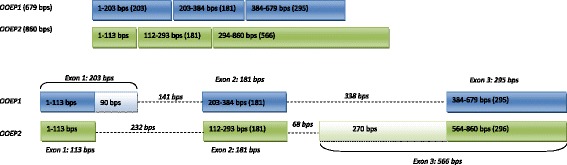
**Alignment of ovine** ***OOEP*** **transcript variant I (cDNA: 679 bps) and variant II (cDNA: 860 bps) (spanning 1126 bps genomic DNA).** Squares indicate exons: the filled parts indicate regions common to both transcripts, while open squares indicate non homologous segments. Dotted line indicate introns.

The full-length cDNA of *OOEP* was cloned by RACE technique in two fragments: the 5′ and the 3′. The amplification of the clones containing the 5′ fragment returned two products of different length (541 and 722 bps), while the amplification of the clones containing the 3′fragment returned one product of 227 bps. In summary, two transcript variants were isolated: one of 679 bps (*OOEP1*) and one of 860 bps (*OOEP2*) (Figure 1). The sequences were submitted to GenBank (http://www.ncbi.nlm.nih.gov/genbank/; accession numbers: KF218578 for *OOEP1* and KF741040 for *OOEP2*).

The alignment of the cDNA sequence with genomic DNA showed that *OOEP1* consists in 3 exons of 203, 181 and 295 bps, spaced by two introns of 141 and 338 bps, while *OOEP2* is composed by 3 exons of 113, 181 and 566 bps, spaced by two introns of 232 and 68 bps (Figure 1).

BLAST analysis of *OOEP1* cDNA confirmed the homology with the orthologous genes present in public databases: ovine sequence shares 95% identity with the bovine (NM_001077869.2; query cover 96%), 81% with the porcine (NM_001198917.1; query cover 56%), 74% with human (NM_001080507.2; query cover 79%) and 72% with the murine (NM_174877; query cover 27%) sequences.

According to *in silico* translation, *OOEP1* encodes a protein of 140 amino acids, and is homologues to the predicted mRNA sequence available in NCBI website (GenBank accession number XM_004011571).

**Figure 2:**
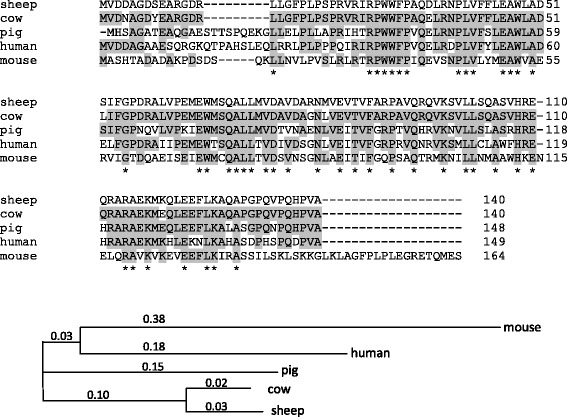
**Multiple alignment of ovine OOEP1 protein sequence with orthologous proteins and construction of phylogenetic neighbour-joining tree (showing distance values) were performed with Clustal Omega (** **http://www.ebi.ac.uk/Tools/msa/clustalo/** **), homology was calculated with BLAST (** **http://blast.ncbi.nlm.nih.gov/Blast.cgi** **).** Ovine sequence shares 95% identity with the bovine (NP_001071337.1; query cover 100%), 67% with the porcine (NP_001185846; query cover 100%), 64% with human (NP_001073976; query cover 100%) and 45% with the murine (NP_080756; query cover 92%) sequences. Homologous sequences are highlighted in grey.

BLAST analysis with homologous proteins showed that ovine sequence shares 95% identity with the bovine (NP_001071337.1; query cover 100%), 67% with the porcine (NP_001185846; query cover 100%), 64% with human (NP_001073976; query cover 100%) and 45 % with the murine (NP_080756; query cover 92%) sequences (Figure 2).

In *OOEP2* case, it was not possible to identify by *in silico* translation and BLAST analysis any product homologous to the sequences currently available in public databases.

### Expression of ovine *NLRP5, KHDC3, TLE6* and *OOEP* in oocytes and early embryos deriving from adult and prepubertal donors

The existence of *KHDC3*, *TLE6* and *OOEP* mRNAs in sheep was confirmed.

**Figure 3:**
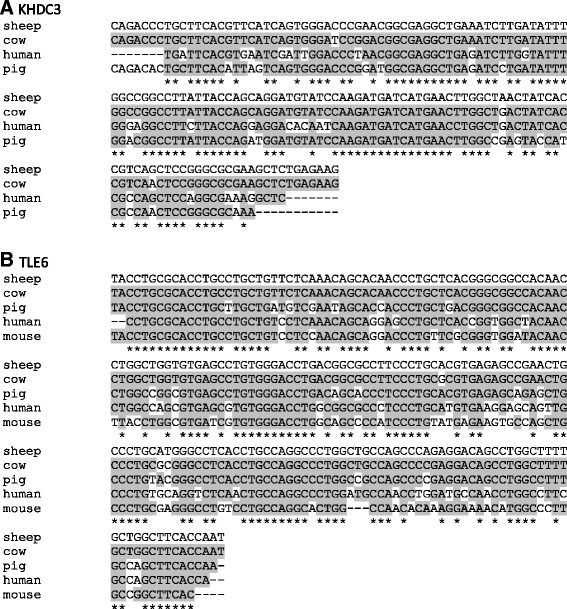
**Fragments of ovine** ***KHDC3L/FILIA*** **and** ***TLE6*** **cDNA sequences and homology with orthologous genes. (A)** The analysed sequence within the *KHDC3L* gene (150 bps) shares 97% homology with the bovine (XM_002690018.2), 86% with the swine (XM_003480279.1) and 82% with human (NM_001017361.2) sequences. **(B)** The analysed sequence within *TLE6* gene (195 bps) shares 98% homology with the bovine (XM_005209025.1), 89% with porcine (XM_003354000.2), 81% with the human (XM_005259645.1) and 78% with murine (NM_053254.2) sequences. Homologous sequences are highlighted in grey.

The sequence of *KHDC3* and *TLE6* fragments is shown in Additional file 1, together with the homology with other mammalian species (Figure 3). The analysed sequence within the *KHDC3* gene (150 bps) shares 97% homology with the bovine (XM_002690018.2), 86% with the swine (XM_003480279.1) and 82% with human (NM_001017361.2) sequences. The analysed sequence within TLE6 gene (195 bps) shares 98% homology with the bovine (XM_005209025.1), 89% with porcine (XM_003354000.2), 81% with the human (XM_005259645.1) and 78% with murine (NM_053254.2) sequences.

Real time PCR analysis confirmed that *NLRP5*, *KHDC3*, *TLE6*, *OOEP1* and *OOEP2* are expressed in ovine oocytes (GV and MII stage) and in vitro produced early embryos (2-cell, 4-cell, 8-cell, 16-cell stage) derived from adult and prepubertal oocyte donors.

**Figure 4:**
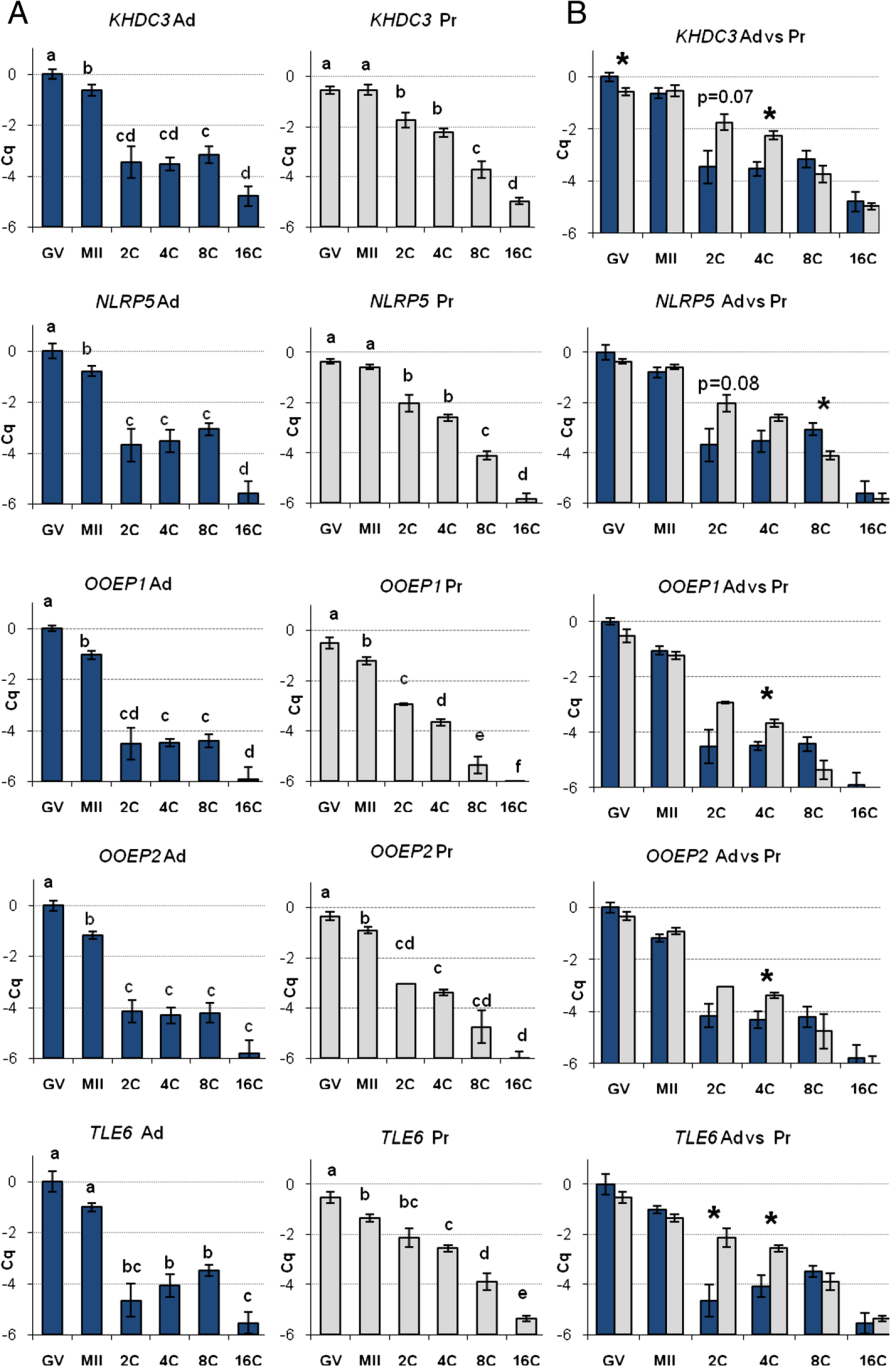
**Relative expression of** ***KHDC3/FILIA*** **,** ***NLRP5/MATER*** **,** ***OOEP1*** **,** ***OOEP2*** **and** ***TLE6*** **in ovine immature (GV) and IVM MII oocytes (MII), in IVMFC two- (2C), four- (4C), eight- (8C), and 16-cell (16C) embryos derived from adult (Ad) or prepubertal (Pr) donors.** Relative abundance values are expressed as ΔCq and show the mean value ± s.e.m. of five (GV and MII) and three (2C, 4C, 8C and 16C) replicates for each stage (each replicate = pool of 10 oocytes/embryos). **A**. Different letters indicate a significant difference in relative mRNA abundance (P <0.05) among the developmental stages. **B**. * indicates a significant difference in relative mRNA abundance (P <0.05) in pairwise comparisons of oocytes/embryos at the same developmental stage derived from adult or prepubertal donors (Ad vs Pr).

**Figure 5:**
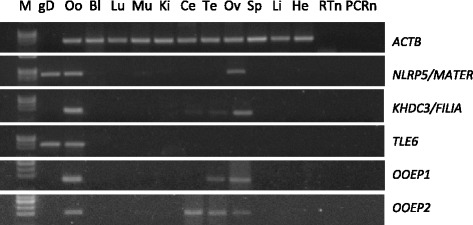
**Tissue-specific expression of** ***NLRP5, KHDC3, TLE6, OOEP1 and OOEP2,*** **determined by total RNA reverse transcription and gene specific PCR. **Actin B was analysed to control RNA integrity. M = marker TrackIt φX174RF DNA/HaeIII, Lane 1 = genomic DNA; cDNA isolated from oocytes (Oo), blastocysts (Bl), lung (Lu), muscle (Mu), kidney (Ki), cerebellum (Ce), testis (Te) ovary (Ov), spleen (Sp), liver (Li) and heart (He) tissues. RTn = negative control, PCRn = PCR negative control.

The expression of all analysed mRNAs appear to be maximal at the GV stage and to decrease during embryo preimplantation development (Figure 4). No transcript was detected at the blastocyst stage (Figure 5).

In oocytes and embryos derived from adult animals, all transcripts show a significant decrease in abundance between GV and MII (except for *TLE6*), between MII and 2C embryos and between 8C and 16C embryos (except for *OOEP2*). In oocytes and embryos derived from Pr, the decreases are less sharp and involve different stages of development compared to Ad: i.e. *NLRP5* abundance is stable during Pr oocyte maturation, conversely to what observed in Ad, but shows a decrease between 4C and 8C Pr embryos, which does not occur in Ad. Similar peculiarities may be observed in *KHDC3*, *OOEP1*, *OOEP2* and *TLE6* expression patterns in Pr (Figure 4). The pair-wise comparison between the expression patterns of Ad vs Pr groups (within each stage separately) reveals similar abundance for all five transcripts in GV and MII oocytes, with the exception of the lower *KHDC3* levels in immaturePr oocytes. Conversely, all analysed mRNAs show significant differences around the 2 and 4 cell stage, with a higher abundance in the Prgroup (Figure 4).

### *OOEP1, OOEP2, TLE6, NLRP5* and *KHDC3* tissue distribution

Semi-quantitative analysis of *NLRP5*, *KHDC3*, *TLE6*, *OOEP1* and *OOEP2* expression in somatic tissues was carried out by PCR. *NLRP5* and *TLE6* are expressed only in ovaries, *KHDC3* in ovaries, faintly in testis and cerebellum. *OOEP1* was found in ovaries and testis, while *OOEP2* in ovaries, testis and cerebellum. No transcripts were detected in the other tissues (Figure 5).

## Discussion

This is the first study that analyses the expression patterns of the SCMC components in a large mammalian species and associates the observed patterns to the developmental potential of the oocyte.

The existence of *TLE6*, *KHDC3* and *OOEP* transcripts in sheep was assessed, and their expression in oocytes, IVP pre-implantation embryos and somatic tissues was characterized, together with the expression of *NLRP5,* whose existence in sheep we had previously reported [24].

A model of differential competence consisting of oocytes derived from adult (Ad) or prepubertal (Pr) donors was used to analyse the expression of SCMC four components in relation to the oocyte developmental ability. Finally, the full length cDNA of *OOEP* was determined by Rapid Amplification of cDNA Ends (RACE).

In mouse, the abundance of the SCMC component mRNAs is maximal in in vivo derived, fully grown oocytes, abruptly decreases during ovulation and disappears by the 2-cell stage [1]. In our observations in sheep, expression of the SCMC components appeared to be maximal in GV oocytes and then to tail off during in vitro oocyte maturation and early embryo development. The transcripts persist in pre-implantation embryos up to the 8–16 cell stage and are not detectable at the blastocyst stage. In the Ad group, significant decreases in abundance are observed during maturation (except *TLE6*), between MII oocytes and 2C stage, and between 8- and 16 cell stage (Figure 4). The differences in expression observed between mouse [1] and sheep are in accordance with the different time in embryonic genome activation (EGA) in the two species. In sheep, the major activation of the embryonic genome takes place later as compared to rodents (e.g. 8-16-cell stage vs. 1-2-cell stage). The spreading of EGA over 3 to 4 cell cycles makes the ovine species an interesting model for studying the transcriptome because it allows a deep analysis of the progressive phases leading to the major wave of transcriptional activation. In fact, while in mouse a single major decrease in SCMC transcript abundance is abruptly observed during ovulation in vivo [1,33], the patterns of expression in sheep highlight three different significant reductions in abundance (during maturation, around fertilization and between 8- and 16 cell stage), suggesting the need for the complex in three different moment of pre-implantation embryo development progression (Figure 4).

We have analysed the expression patterns of the SCMC in a model of differential developmental competence, in order to verify a potential link between the mRNA abundance and the quality of the gamete. The model consists in ovine oocytes deriving from adult versus prepubertal donors; the oocytes derived from prepubertal animals can be fertilized, develop and give rise to a live offspring, however they show significanty lower developmental competence compared to oocytes derived from adult donors [34-36]. Both cytoplasmic and nuclear maturation of the oocyte are essential for the formation of an egg having the capacity for fertilization and development to live offspring [37]. Nuclear maturation encompasses the processes reversing meiotic arrest at prophase I and driving the progression of meiosis to metaphase II. Cytoplasmic maturation refers to the processes that prepare the egg for activation and preimplantation development [37]. The reduced developmental ability shown by Pr oocytes indicates that they are competent to undergo nuclear maturation, but contain a subset of molecules sufficient to support development only to some extent [38]. Therefore, Ad and Pr represent gametes of high versus low developmental competence, respectively, and constitute an ideal model for studying the relationship between cytoplasmic and nuclear competence of the oocyte. Interestingly, the expression analysis of the SCMC components in Ad vs Pr model consistently highlighted significant differences around the two-four cell stage of embryo development, with higher abundance in the Pr group (Figure 4). Although the exact function of the SCMC is yet to be clarified, the persistence of the transcripts until the 8–16 cell stage presumes the need of the complex around oocyte to embryo transition and possibly beyond. The observation of similar levels of mRNAs in the Ad and Pr oocytes together with a higher abundance in the Pr 2C-4C embryos suggests a delay in the use of the transcripts by the Prembryos. The decrease in abundance observed in Ad embryos may be due to translation of the transcripts, in case more SCMC is needed, or to their degradation, if synthesis of the complex is no longer necessary. In Pr, however, we observed a delay in the decrease of SCMC component transcripts during early embryo development, just before major genome activation of the nascent embryo. Whether the delay in the Pr embryos causes a shortage of SCMC or a surplus of transcripts, this may be detrimental for the progression of embryo development. The regulation of maternal transcript degradation is indeed as important as the accumulation of these molecules during oocyte growth, and the disruption of any of these mechanisms may severely impair the oocyte developmental competence [39]. Further analysis at protein level will clarify whether the SCMC protein content in oocytes and embryos reflects the observed transcriptional picture.

*OOEP/FLOPED* and *KHDC3/FILIA* belong to a family of genes with several members, located in a single syntenic region, that encode structurally related proteins with an atypical RNA-binding N-terminal KH domain, named FILIA N Like, and are specifically expressed in oocytes and ⁄ or embryonic stem cells [27]. Compared to other canonical KH domains, a conserved N-terminal extension is identified prior to the KH domain in these proteins, although their function remains unknown. Interestingly, they are absent from fish, bird, and marsupial genomes and thus seem to have first appeared in eutherian mammals, in which they have evolved rapidly.

Two different research groups independently identified murine *Ooep*/*Floped* as a maternal effect gene, and part of the SCMC [1,3]. Maternally derived OOEP protein was seen to be essential for normal first cleavage and subsequent cell divisions: *Ooep*-null female mice are completely infertile because of a developmental impairment of the preimplantation embryos, that show slower cleavage and asymmetric blastomeres at 2 cell stage and never progress beyond [3]. These results indicate that OOEP protein has an important function around the zygotic stage, when mouse EGA occurs. In sheep, this corresponds to the 4–8 cell stage; as a consequence, the alteration in *OOEP* transcript abundance we observed in ovine embryos at the 2–4 cell stage would likely affect proper development.

We determined the sequence of the full-length *OOEP* cDNA from ovine ovary using rapid amplification of cDNA ends (RACE) and found two transcript variants of different length (679 and 860 bps). The shorter variant, hereafter referred to as *OOEP1* (GenBank accession number: KF218578), was seen to be identical to the predicted full-length cDNA present in the NCBI database, with accession number XM_004011571. According to *in silico* translation, *OOEP1* encodes for a 140 amino acid peptide that contains the atypical FILIA N Like domain, shared by the members of the above mentioned, rapidly evolving family of oocyte specific genes [27]. In accordance, the homology of the ovine gene with other mammalian species ranges from 95% identity in a 96% query cover with Bos taurus, to the 72% of only 27% query cover with M. Musculus.

Conversely, the second variant (*OOEP2;* GenBank *a*ccession number KF741040) is not homologous to any EST found in the NCBI database, and its potential reading frame is not obvious. The same reading frame for the two variants would be suggested by the exon-intron structure of the two transcripts. As shown in Figure 1, the second exon is identical in two variants, while the first and the third ones differ. The two non-homologous segments of exon 1 and 3 are 90 and 270 bps long, respectively; they display the characteristics of “symmetrical introns” [40] because their length is an exact multiple of 3, so they potentially do not shift the reading frame. Nevertheless, applying to *OOEP2* the same reading frame as *OOEP1*, a premature termination codon (PTC) lying in exon 1 stops its translation after 36 amino acids. The expression analysis of *OOEP* during oocyte maturation and embryo development showed that both transcripts persist with similar decreasing abundance up to the 16 cell stage (Figure 4). However, in the hypothesis of the same reading frame as *OOEP1*, the presence of a PTC would prevent the second variant from being translated.

Alternative splicing is known to produce also splice forms that are not being translated into proteins, but rather play a regulatory role [10,41]. It was suggested that as much as 35% of human alternative isoforms contain PTCs [42]; this widespread “unproductive” alternative splicing implies an important regulatory role, probably a post-transcriptional control of distinct genes [42-44]. A number of these non-protein coding transcripts are clearly processed from pre-mRNAs, and their genomic structure encompasses an exon–intron organization [10]. In this scenario, a regulatory role, possibly gene-specific, may be hypothesized for *OOEP2*.

A picture similar to *OOEP* transcription in sheep was observed for *Khdc3/Filia* in mouse oocytes and embryos: *Khdc3* is transcribed as two types of transcripts with respective lengths of 1.2 k and 1.6 k base pairs that co-exist in oocytes, but only the former transcript is translated into a functional protein with 346 residues [33].

The identification of the alternative noncoding *OOEP* gene transcript, *OOEP2*, in ovine oocytes adds sheep to the list of mammalian species with experimentally confirmed observation of aberrant splicing during oogenesis.

In mouse, maternal KHDC3 plays a role in maintaining euploidy in cleavage-stage embryogenesis by integrating proper spindle formation; *Khdc3* knock out females are not completely sterile, but their early embryos exhibit significant delays in development in association with lagging chromosomes, micronuclei and aneuploidy [4]. KHDC3 was seen to form a stable dimer in solution and to bind polynucleotides and endogenous RNA in vitro [45]. The atypical KH domain in *Khdc3* and *Ooep* gene family binds RNA like other canonical KH-domain proteins, but might have unique consequences that are restricted to oocytes and early embryonic development [45]. Due to their similar expression profiles and their versatile binding with RNA via KH domains, [45] speculated that KHDC3 and OOEP function, either as a homo-dimer or in a multiple-KH manner together in the SCMC in vivo, in RNA degradation during oocyte maturation or early embryogenesis. Our analysis in sheep confirms a similar pattern of expression for the two genes in ovine oocytes and pre-implantation embryos, and the presence of the atypical FILIA N Like domain in the protein deduced from the sequence of ovine *OOEP1* transcript; as a consequence, a similar role may be hypothesized in the ovine species as well.

In human, controversial evidence exists on a potential association between mutations in *KHDC3* and recurrent biparental complete hydatidiform mole, which is the only known pure maternal-effect recessive inherited disorder in humans that results in recurrent pregnancy losses [46]. Mutations in *KHDC3* were found in women with familial diploid biparental hydatidiform mole s from four families [47,48] and in two unrelated women with diploid biparental hydatidiform mole s but without familial hydatidiform mole s [49]. However, discordant evidence seems to exclude mutations in this gene as a cause of non-familial biparental moles [50] and androgenetic moles [51].

The analysis of the SCMC component expression in somatic tissues pointed out additional information on the differences and overlaps between sheep and mouse species. In mouse, all four genes are expressed predominantly in the ovary [1,8,33]. In sheep, all genes are expressed in ovaries as well, but not exclusively: *KHDC3* mRNA was detected also in testis and cerebellum, albeit faintly, while *OOEP* transcript variants interestingly display two different patterns: *OOEP1* was found in ovaries and testis whereas *OOEP2* is expressed also in cerebellum (Figure 5). No transcripts were detected in the other tissues. This is in accordance with the observation of *NLRP5/MATER* expression in mouse and swine [8,25], while bovine *NLRP5* was observed also in testis [52]. No information on the expression of *TLE6, KHDC3* and *OOEP* in somatic tissues of species other than mouse is available. Furthermore, while no SCMC transcripts were detected in ovine blastocysts, murine *Khdc3* is expressed also by the embryo at the morula and blastocyst stages [33].

The expression of a MEG in nervous tissue has been previously reported in the case of *Fmn2*, a MEG expressed in the developing mammalian oocyte and required for DNA-spindle positioning during meiosis I [53]. The *Fmn2* genes in mouse and humans share conservation of sequence and genomic location, and were seen to be expressed throughout the brain and spinal cord [54].

## Conclusions

In summary, this work describes for the first time the expression of the SCMC components in oocytes and in vitro produced preimplantation embryos in sheep. It reports the association between the patterns of expression in early embryos and the oocyte developmental potential in terms of delayed degradation of maternal *NLRP5*, *KHDC3*, *OOEP* and *TLE6* transcripts in incompetent oocytes.

By reporting the alternative noncoding *OOEP* gene transcript, *OOEP2*, it adds sheep to the list of mammalian species with experimentally confirmed observation of aberrant splicing during oogenesis.

The analysis of SCMC component expression patterns in a large mammalian species highlighted species-specific features that advance our understanding on the molecular differences underlying cytoplasmic vs nuclear maturation of the oocytes, and subsequent early embryonic development in mammals. Further analysis at protein level will clarify whether the observed transcriptional picture reflects the SCMC protein content in oocytes and embryos.

The comprehension of dissimilarities and overlaps in transcriptome composition between model organisms provides valuable information on genes that may play important regulatory roles in fertility in nonmurine models, including the human.

## Additional file

Additional file 1:
**Fragments of ovine** ***KHDC3/FILIA*** **and** ***TLE6*** **cDNA sequences.** (a) Analysed sequence within the *KHDC3* gene (150 bps). (b) Analysed sequence within *TLE6* gene (195 bps).
