# Supplementary material for: Expression of maternally derived KHDC3, NLRP5, OOEP and TLE6 is associated with oocyte developmental competence in the ovine species
Source: BMC Dev Biol. 2014 Nov 25;14:40. doi: 10.1186/s12861-014-0040-y (PMC4247878; doi:10.1186/s12861-014-0040-y)
Supplement: Additional file 1: — Fragments of ovine KHDC3/FILIA and TLE6 cDNA sequences. (a) Analysed sequence within the KHDC3 gene (150 bps). (b) Analysed sequence within TLE6 gene (195 bps). [file 12861_2014_40_MOESM1_ESM.pdf]

Additional file 1.
Fragments of ovine *KHDC3/FILIA* and *TLE6* cDNA sequences.
(a) Analysed sequence within the *KHDC3* gene (150 bps).
(b) Analysed sequence within *TLE6* gene (195 bps).

a) *KHDC3*

```
CAGACCCTGCTTCACGTTCATCAGTGGGACCCGAACGGCGAGGCTGAAATCTTGATATTT
GGCCGGCCTTATTACCAGCAGGATGTATCCAAGATGATCATGAACTTGGCTAACTATCAC
CGTCAGCTCCGGGCGCGAAGCTCTGAGAAG
```

b) *TLE6*

```
TACCTGCGCACCTGCCTGCTGTTCTCAAACAGCACAACCCTGCTCACGGGCGGCCACAAC
CTGGCTGGTGTGAGCCTGTGGGACCTGACGGCGCCTTCCCTGCACGTGAGAGCCGAACTG
CCCTGCATGGGCCTCACCTGCCAGGCCCTGGCTGCCAGCCCAGAGGACAGCCTGGCTTTT
GCTGGCTTCACCAAT
```
